# Gender Differences in Caregiving of Older Adults: A Systematic Review of the Literature

**DOI:** 10.1093/geroni/igaa057.491

**Published:** 2020-12-16

**Authors:** Michael Bueno, Jo-Ana Chase

**Affiliations:** 1 University of Missouri - Columbia, Columbia, Missouri, United States; 2 University of Missouri, Columbia, Columbia, Missouri, United States

## Abstract

Caregivers of older adults with chronic illness provide essential care that benefits individuals and society. Many factors influence health outcomes related to the caregiving role. The purpose of this review is to explore how caregiver health outcomes may vary by gender. Data sources include CINAHL, Google Scholar, Google and PsychINFO. Eligible studies focused on gender-based differences in psychological and emotional outcomes of primary informal or familial caregivers (ie, spouses, children, grandchildren, nieces, nephews, neighbors, and friends) of an older adult with chronic illness. The Caregiver Identity Theory (CIT) guided this study. Twelve studies were eligible for this review. Consistent with the CIT, negative outcomes are associated with changing role identity throughout the care recipient’s disease progression, and these outcomes differ between genders. Women tended to experience higher overall burden, stress, anxiety, shame and role strain; however, some studies found that men may be affected more during the initial transition into the caregiver role. Methodological limitations of primary research in this area included the use of cross-sectional design, poor generalization to US populations since most studies were conducted outside of the US, and disproportionately fewer male participants across studies. Overall, gender differences in health outcomes among caregivers exist, and other variables relating to gender, such as kinship and age, may also be important factors. Findings suggest health professionals should create individualized engagement, communication, and training strategies with considerations for gender differences in design. Future studies including more male caregivers and more diverse participants is warranted.

